# Supplementary material for: Synergistic aluminum dual-atom sites and nickel nanoclusters for acetylene selective hydrogenation
Source: Nat Commun. 2026 Mar 6;17:3542. doi: 10.1038/s41467-026-70323-4 (PMC13086864; doi:10.1038/s41467-026-70323-4)
Supplement: Supplementary file 1 — Supplementary Information [file 41467_2026_70323_MOESM1_ESM.pdf]

Synergistic Aluminum Dual-Atom Sites and Nickel Nanoclusters for Acetylene Selective hydrogenation

Yanan Liu[1,2,δ], He Yu[1,δ], Mengjiao Li[3,δ], Li Yan[1,δ], Ruihu Lu[3], Xiuting Fu[1], Zhenfei Zhang[1], Youqi Zhu[4], Ziyun Wang[3,*], Shubo Tian[1,2,*]

[1]State Key Laboratory of Chemical Resource Engineering, Beijing Engineering Center for Hierarchical Catalysts, Beijing University of Chemical Technology, Beijing, 100029, China

[2]Quzhou Institute for Innovation in Resource Chemical Engineering, Quzhou 324000, China

[3]School of Chemical Sciences University of Auckland, 1010 Auckland, New Zealand

[4]Beijing Key Laboratory of Construction Tailorable Advanced Functional Materials and Green Applications, School of Materials Science and Engineering, Beijing Institute of Technology, Beijing, China.

* Corresponding author

E-mail address: ziyun.wang@auckland.ac.nz (Z. W.); tianshubo@mail.buct.edu.cn (S. T.)

Supplementary Table 1. Elemental content determined by ICP analysis for different catalysts.

Catalysts	Al (%)	Ni (%)
Al_2-Ni_{NC}/NCNT	0.43	0.28
Al_2/NCNT	0.45	-
Al_1/NCNT	0.42	-
Ni_{NC}/NCNT	-	0.31

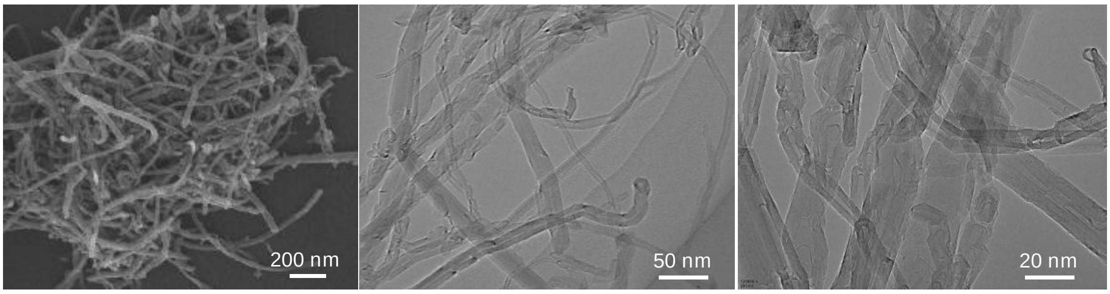

Supplementary Fig. 1 SEM and HRTEM images to explore the morphology and dispersion of Al_2/NCNT catalyst

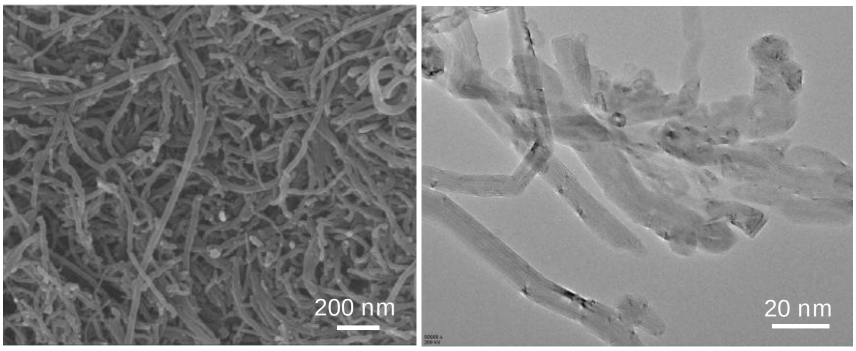

Supplementary Fig. 2 SEM and HRTEM images to explore the morphology and dispersion of Al_2-Ni_{NC}/NCNT catalyst

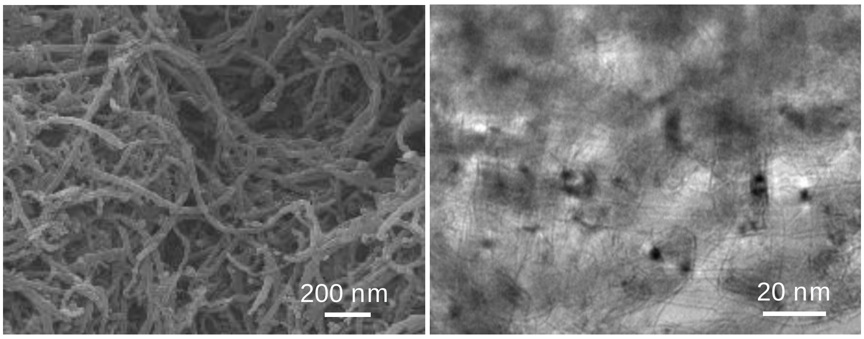

Supplementary Fig. 3 SEM and HRTEM images to explore the morphology and dispersion of Ni_{NC}/NCNT catalyst

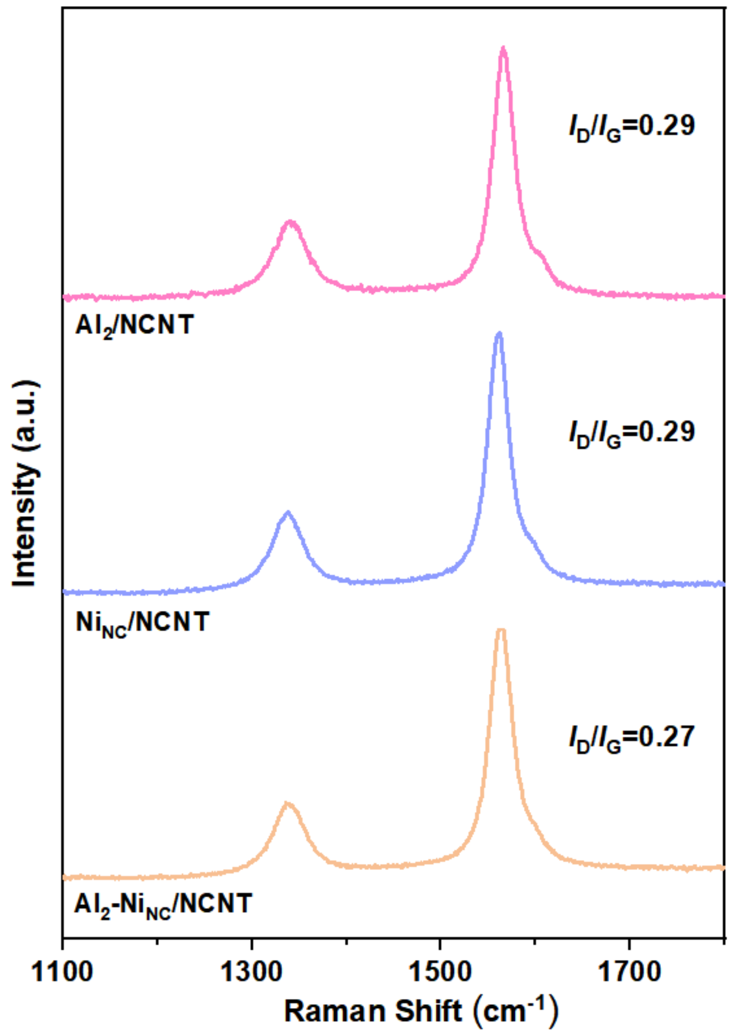

Supplementary Fig. 4 Raman spectra of NCNT loaded different active species to analyze the property change of NCNT support

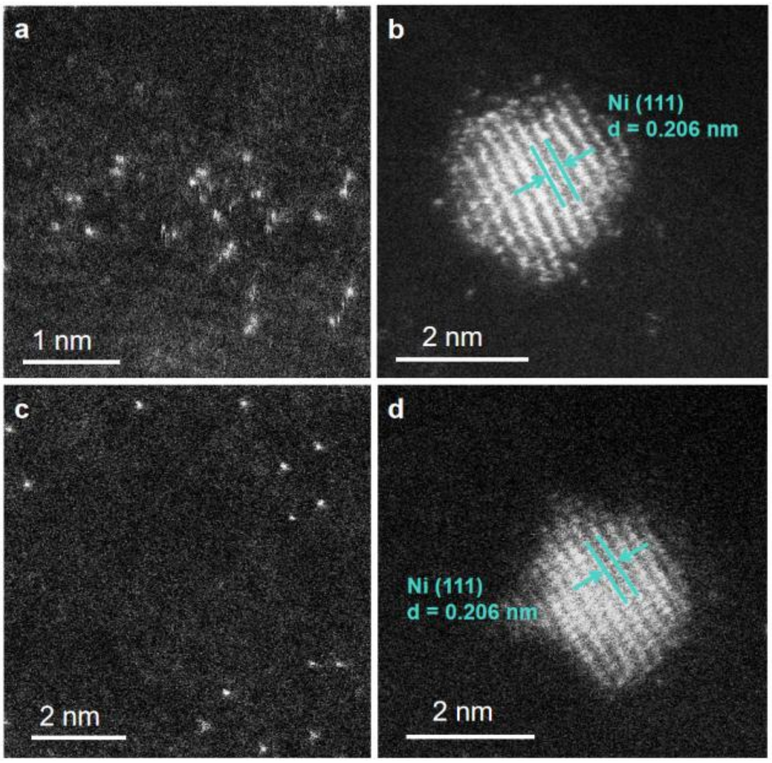

Supplementary Fig. 5 Atom distribution of different catalysts. Aberration-corrected HAADF-STEM images of (a) Al_2/NCNT (b) Al_2-Ni_{NC}/NCNT (c) Al_1/NCNT without the circles and (d) Ni_{NC}/NCNT

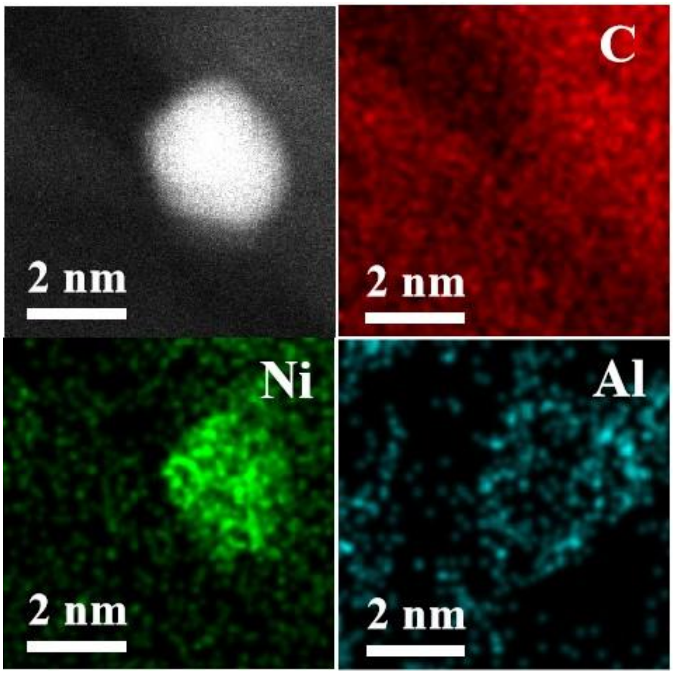

Supplementary Fig. 6 Atom distribution of Ni and Al species in Al_2-Ni_{NC}/NCNT catalyst derived from the EDX element mapping

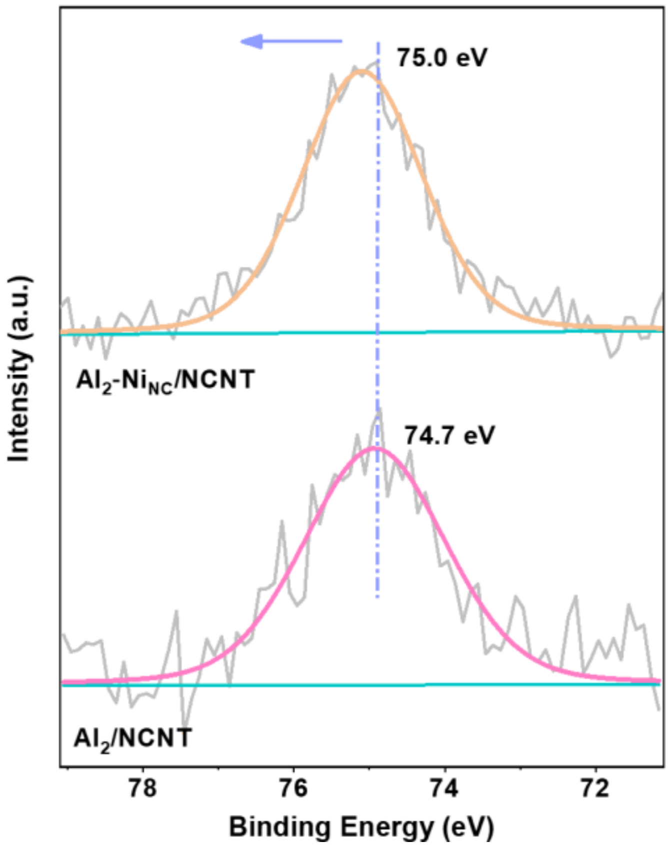

Supplementary Fig. 7 Analysis of electronic structure. Al 2*p* XPS spectra of Al_2-Ni_{NC}/NCNT and Al_2/NCNT

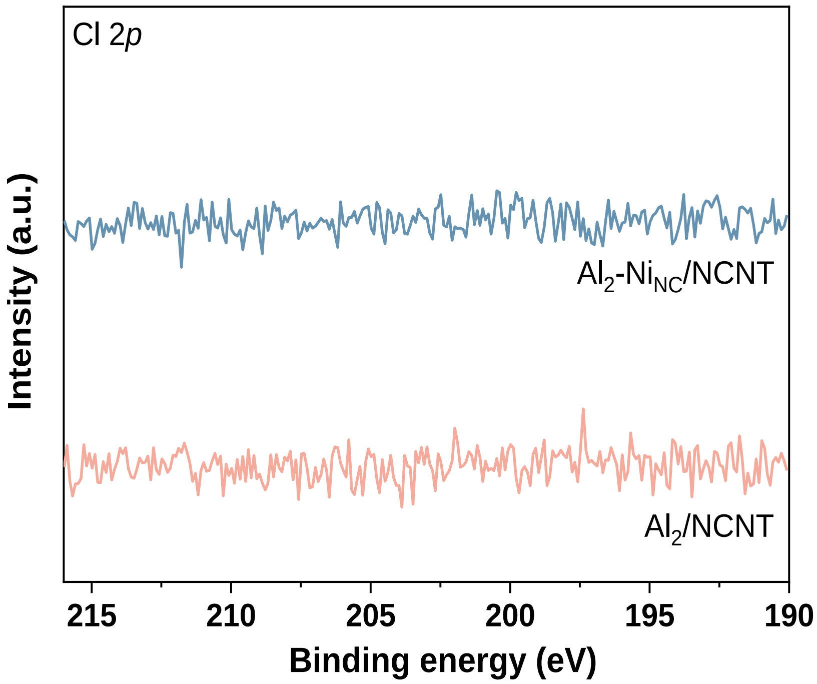

Supplementary Fig. 8 Analysis of Cl species in Al_2-Ni_{NC}/NCNT and Al_2/NCNT using Cl 2*p* XPS spectra

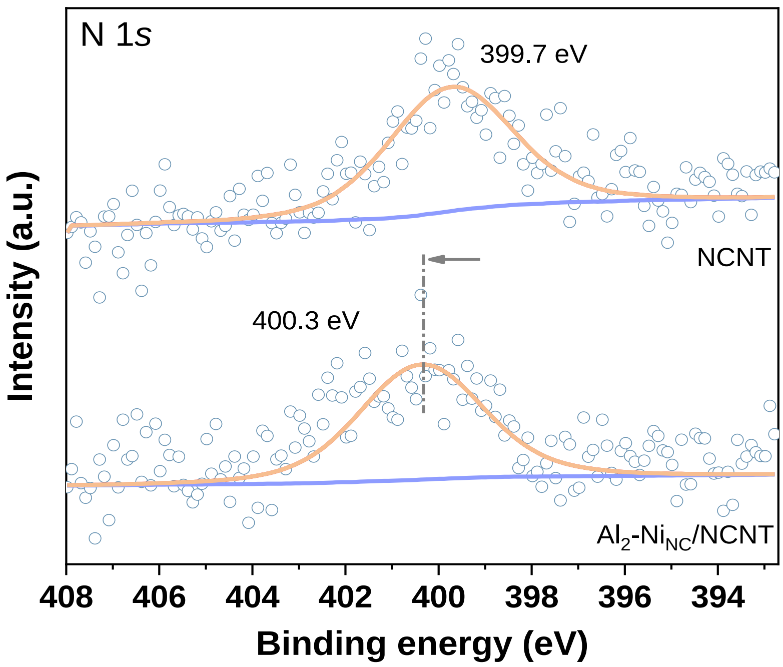

Supplementary Fig. 9 Analysis of electronic structure. N 1*s* XPS spectra of Al_2-Ni_{NC}/NCNT and NCNT

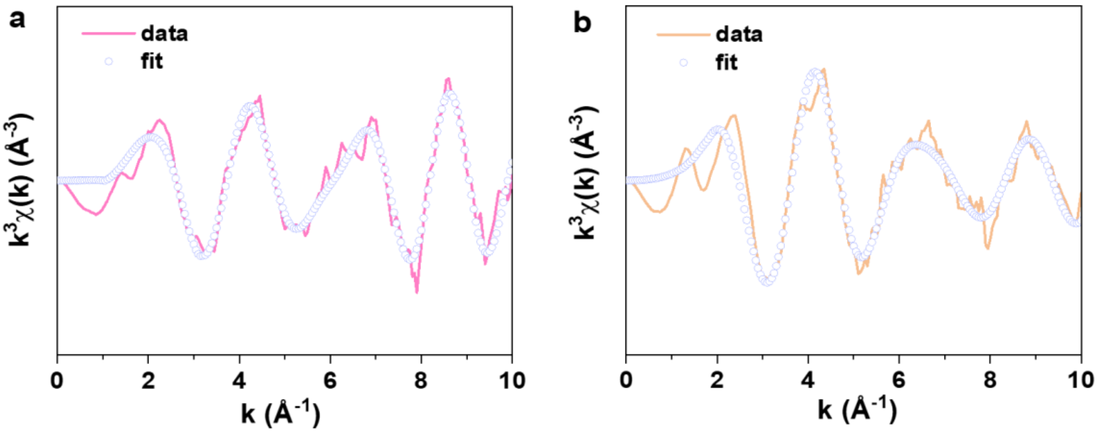

Supplementary Fig. 10 Analysis of coordination environment for Al species. Al K-edge EXAFS fitting curves in k space over (a) Al_2/NCNT and (b) Al_2-Ni_{NC}/NCNT

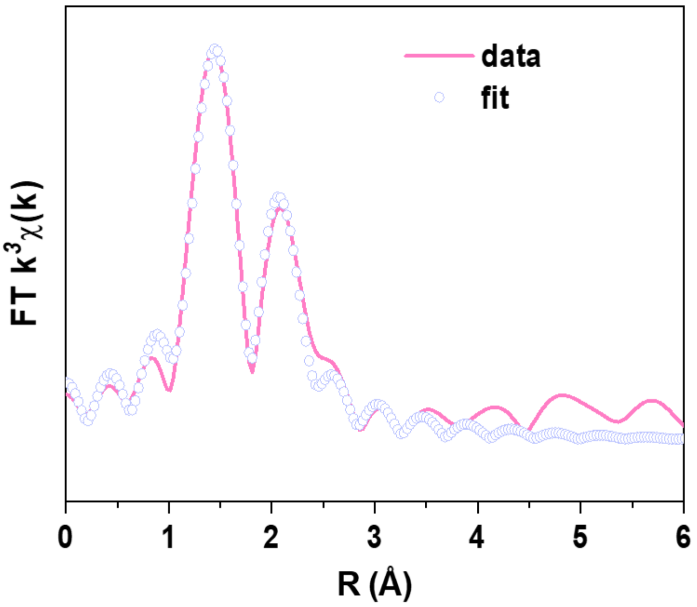

Supplementary Fig. 11 Analysis of coordination environment for Al species. Al K-edge EXAFS fitting curves in R space over Al_2/NCNT

Supplementary Table 2. Al K edge EXAFS fitting results of samples.

Sample	Shell	*CN*[a]	*R*(Å)[b]	σ^2 (Å^2)[c]	ΔE_0(eV)[d]	*R* factor
Al_1/NCNT	Al-N	4.2 ±0.5	1.82 ±0.02	0.0051	3.5	0.02
Al_2/NCNT	Al-N	3.8 ±0.5	1.80 ±0.02	0.0041	4.5	0.01
	Al-Al	1.1 ±0.4	2.44 ±0.02	0.0095		
Al_2-Ni_{NC} /NCNT	Al-N	3.9 ±0.4	1.78 ±0.02	0.0039	-2.9	0.01
	Al-Al	0.9 ±0.5	2.32 ±0.02	0.0023		

[a] *CN*, coordination number; [b] *R*, atom distance; [c] σ^2, Debye-Waller factor to account for both thermal and structural disorders; [d] ΔE_0, inner potential correction; *R* factor indicates the goodness of the fit.

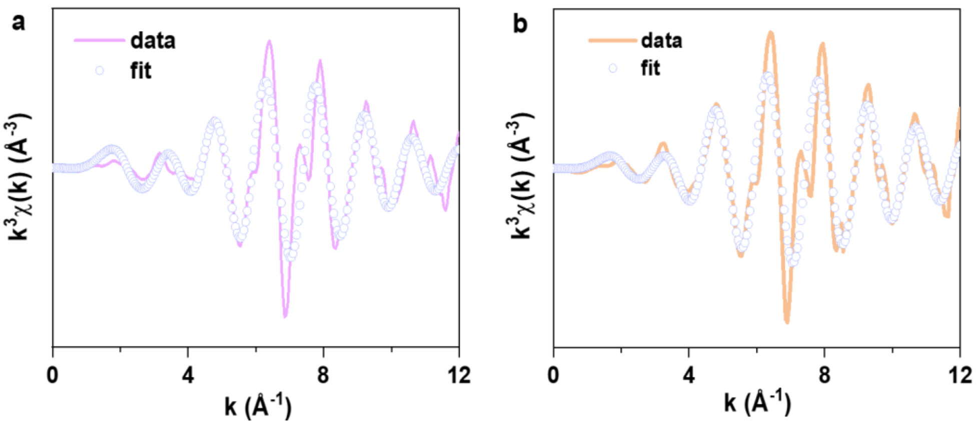

Supplementary Fig. 12 Analysis of coordination environment for Ni species. Ni K-edge EXAFS fitting curves in k space over (a) Ni_{NC}/NCNT and (b) Al_2-Ni_{NC}/NCNT

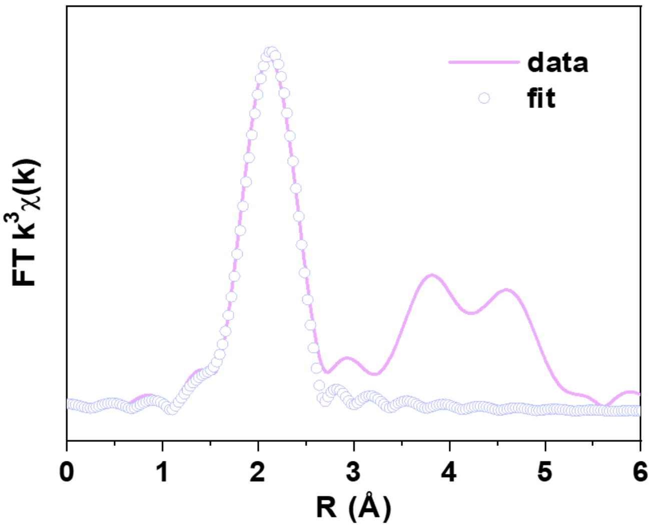

Supplementary Fig. 13 Analysis of coordination environment for Ni species. Ni K-edge EXAFS fitting curves in R space over Ni_{NC}/NCNT

Supplementary Table 3. Ni K edge EXAFS fitting results of samples.

Sample	Shell	CN^a	R(Å)^b	σ^2 (Å^2)^c	ΔE_0(eV)^d	R factor
Ni_{NC}/NCNT	Ni-N	2.1±0.5	2.07±0.02	0.0032	-2.2	0.01
	Ni-Ni	9.7±2.3	2.51±0.02	0.0069		
Al_2-Ni_{NC} /NCNT	Ni-N	0.9±0.2	2.05±0.02	0.0038	-4.8	0.01
	Ni-Ni	10.0±0.6	2.48±0.02	0.0058		

^{a} *CN*, coordination number; ^{b} *R*, atom distance; ^{c} σ^2, Debye-Waller factor to account for both thermal and structural disorders; ^{d} ΔE_0, inner potential correction; *R* factor indicates the goodness of the fit.

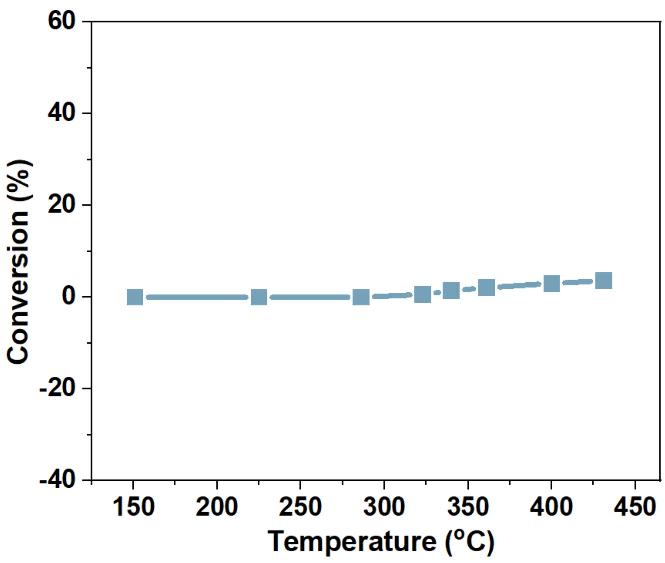

Supplementary Fig. 14 Acetylene conversion of the NCNT

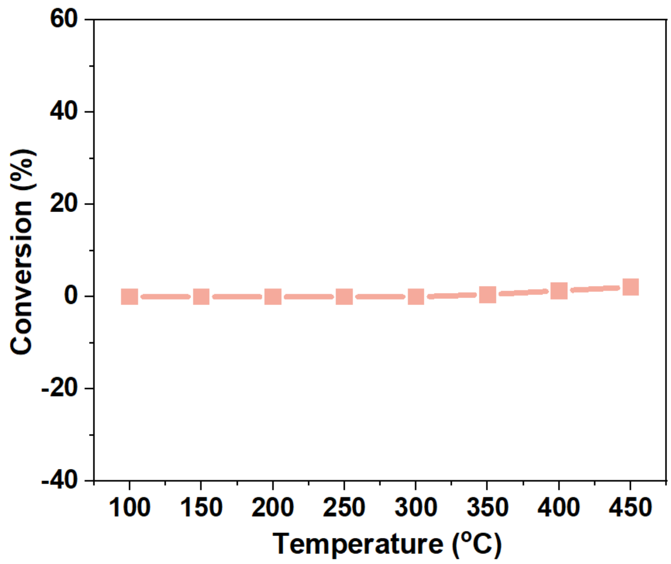

Supplementary Fig. 15 Acetylene conversion of reactor system

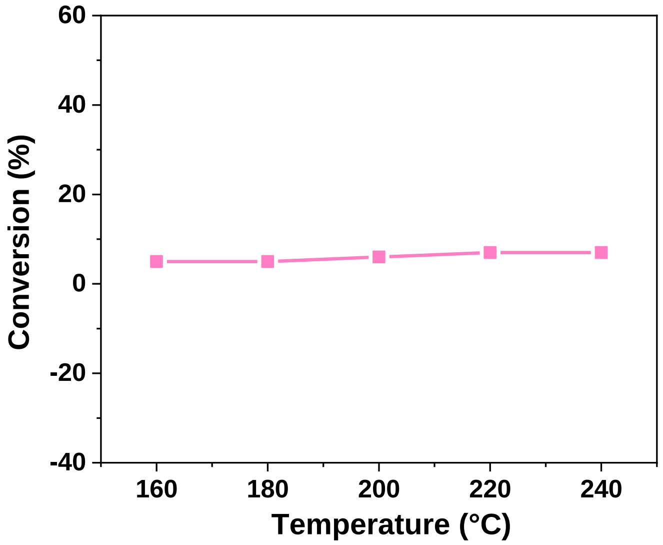

Supplementary Fig. 16 Acetylene conversion of the Al_2/NCNT catalysts

Supplementary Table 4. Orthogonal experiments of Al_2-Ni_{NC}/NCNT at different conditions

Flow rates (mL h^{-1})	Reaction temperature (°C)	H_2/C_2H_2	Conv. (%)	Sel. (%)
2400	157	20:1	81	94.5
1800	157	5:1	26	99.1
1800	157	10:1	59	94.4
1800	157	20:1	100	89.6
1800	157	30:1	100	70.8

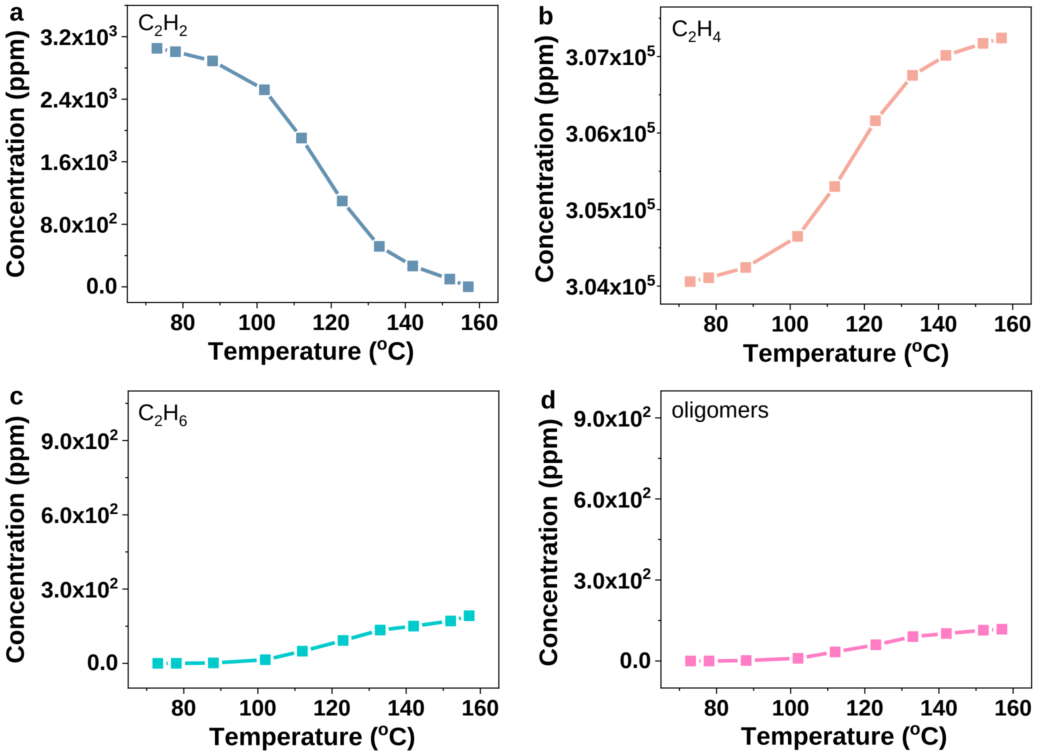

Supplementary Fig. 17 Catalytic performance. The concentrations of (a) acetylene, (b) ethylene (c) ethane and (d) oligomers as a function of temperature over Al_2-Ni_{NC}/NCNT

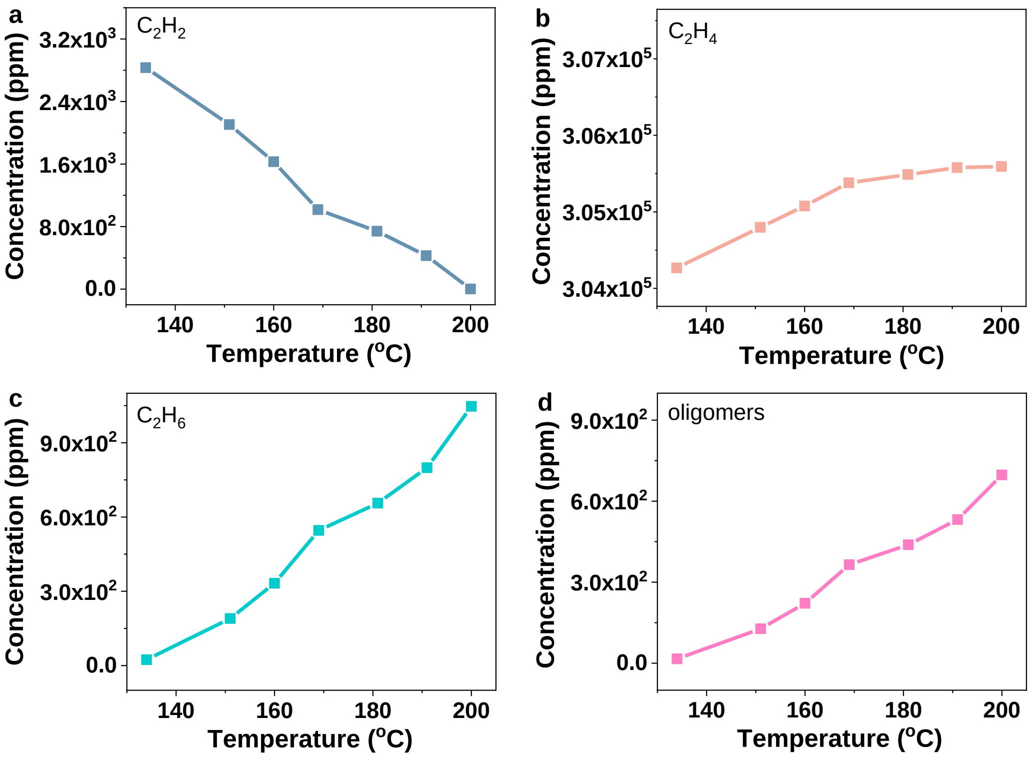

Supplementary Fig. 18 Catalytic performance. The concentrations of (a) acetylene, (b) ethylene (c) ethane and (d) oligomers as a function of temperature over Ni_{NC}/NCNT

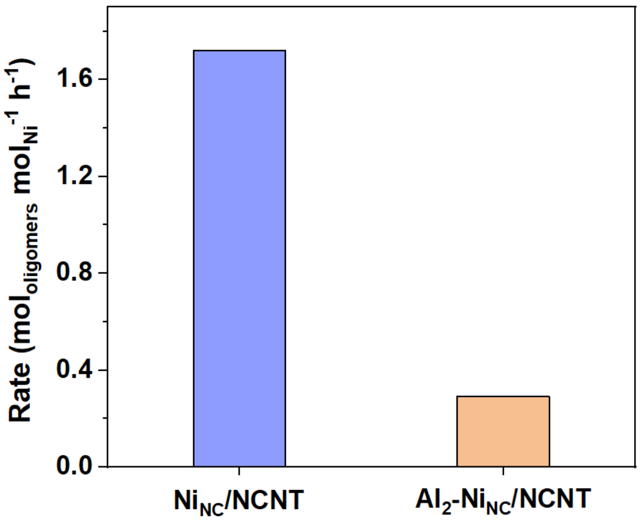

Supplementary Fig. 19 Catalytic performance. The formation rates of oligomers over Ni$_{NC}$/NCNT and Al$_2$-Ni$_{NC}$/NCNT at complete acetylene conversion

Supplementary Table 5. Performance of Ni-based catalysts reported in references

Catalysts	Loading (%)	M (mg)	C_2H_2:H_2:C_2H_4 (vol.%)	T (℃)	Conv. (%)	Sel. (%)
Al_2-Ni_{NC}/NCNT	0.28	0.56	1: 20: 99	157	99.98	90.26
Ni_1Cu_2/g-C_3N_4[1]	3.1	4.65	0.5: 5: 25	170	100	90
NiGa[2]	10	5	1: 10: 20	180	70	75
NiCu/MMO[3]	10	10	0.33:0.66:34.5	160	100	70
Ni-Cu/r-Al_2O_3[4]	4.7	14.1	1: 10: 99	135	100	86
Ni@CeO_2[5]	1.5	-	0.9: 1.9: 33.15	200	65	100
$Ni_3ZnC_{0.7}$/OCNT[6]	5	0.5	0.5: 4.5: 20	200	99	94
$AgNi_{0.5}$/SiO_2[7]	1.09	0.327	1: 20: 79	220	99	65
$CuNi_{0.125}$/SiO_2[7]	0.41	0.123	1: 20: 0	300	100	60
Ni/g-C_3N_4-T[8]	1.31	0.655	1: 10: 20	175	100	84
NiCu/ZrO_2[9]	4.6	1.15	0.5: 5: 25	170	98	86
NiCuFeGaGe/SiO_2[10]	1	2	1: 10: 10	220	100	93
Ni_n/ND@G[11]	0.56	0.336	1: 10: 20	190	100	85
Na-Ni@CHA[12]	3.5	7	0.5: 8: 5	180	100	97
Ni_1MoS/Al_2O_3[13]	3.5	17.5	0.15: 3.03:15	125	100	90
Ni_1Sb_2[14]	10.7	32.1	0.5: 2.5: 30	260	100	93

Supplementary Table 6. Performance of Cu-based and Fe-based catalysts reported in references

Catalysts	Loading (wt%)	M (mg)	C_2H_2:H_2:C_2H_4 (vol.%)	T (℃)	Conv. (%)	Sel. (%)
CuZn/NC2[15]	1.15	-	0.33:0.66:33	180	97	97.5
Cu_1/C_3N_4[1]	8.1	12.15	0.5: 5: 25	200	<10	<60
Cu_1/ND@G[16]	0.5	0.5	1: 10: 20	200	100	93
Cu/Al_2O_3[17]	0.5	4	1: 10:50	188	100	91
Cu_1/ND@G[18]	0.25	0.5	1: 10: 20	200	95	98
$Cu/Fe_{0.16}MgO_x$[19]	8.29	16.58	0.33: 1.02: 32.86	215	100	95
$CuPd_{0.006}/SiO_2$[20]	4.96	1.488	1:20:20	160	100	85
$CuBi/SiO_2$ (HTR)[21]	-	-	0.5:10:20	100	100	91.1
$Cu(OH)_2$[22]	-	-	0.4:10:88.88	110	100	51
Cu_2O[23]	-	-	-	190	100	50.8
Cu/ZrO_2[24]	8.5	2.125	1:10:20	220	15	100
Fe-MOF[25]	2.82	0.705	1.2[a]	150	>99.917	>90

[a] acetylene concentration in an ethylene (1 mL/min, 2 bar) and H_2 (2 mL/min, 4 bar) flow

Supplementary Table 7. Performance of Pd-based catalysts reported in references

Catalysts	Loading (%)	M amount (mg)	C_2H_2:H_2:C_2H_4 (vol.%)	T (℃)	Conv. (%)	Sel. (%)
Pd/Al_2O_3[26]	2.58	1.032	1: 2: 99	138	100	88
Pd_1Cu_1/ND@G[27]	0.09	0.027	1: 10: 20	110	100	92
Pd_1/TiO_2[28]	0.146	0.0219	1: 10: 20	120	100	>50
Pd_1Au_1@[29]	2.9	0.87	1.1: 9.1: 89.8	100	100	89
Pd_1@Cu-SiW[30]	0.41	1.23	0.5: 5: 50	120	100	93
Pd_8Zn_{44}[31]	7.8	1.17	1: 18: 31	160	100	90
$Pd/Bi_2O_3/TiO_2$[32]	2.5	0.75	1: 20: 20	44	90	91
Pd_1/N-graphene[33]	2.3	1.15	1: 20: 20	125	99	94
B2 CuPd[34]	9.5	1.9	0.5: 5: 10	90	100	95
Pd_1/ND@G[35]	0.11	0.033	1:10:20	180	100	90
PdZn-1.2@ZIF-8C[36]	0.1	0.1	0.65:5:50	120	85	80
$PdIn/MgAl_2O_4$[37]	2	0.5	0.5:5:50	90	96	92
Pd@SOD[38]	0.099	0.099	1:10:0	150	99.8	94.5
Pd@C/CNT[39]	1.5	0.075	0.5:3:20	150	93	70
Pd_2Sn/C[40]	0.97	2.716	1:2:99	160	97.5	91
Pd_4S/CNTs[41]	1	0.5	1:1.8:9	200	100	85
$PdAg/TiO_2$[42]	0.64	0.64	1:5:20	95	99	60
Pd-Fe_3O_4-H[43]	-	-	1:6:0	85	100	80
ISA-Pd/MPNC[44]	0.0433	0.0866	0.5:5:50	120	100	80
Pd-SAs @ZIF-8[45]	0.16	0.00256	0.5:5:50	120	96	93.4
Pd_1/C_3N_4[46]	0.5	-	0.5:1:25	115	99	83
$Pd/Ni(OH)_2$[47]	0.005	-	0.65:5:50	105	80	70
Pd/MgO[48]	0.05	0.05	1:2:0	200	100	82
Pd/MgO[49]	0.16	0.032	1:10:20	140	100	70
Pd_1/ZnO[50]	0.01	50	2:20:40	100	100	80
Pd_1/CeO_2[51]	0.098	-	2:20:40	160	100	85
$Pd/MgAl_2O_4$[52]	0.1	0.01	1:5:20	120	96	87

Pd_1-N_8/CNT[53]	0.0092	0.0046	2:4:50	40	83	98
Pd_3/GDY[54]	0.4	-	0.33:0.66:32.8	160	100	97
PdZn/ZnO[55]	0.7	0.7	2:20:40	80	92	89
PdAg/Al_2O_3[56]	0.0003	-	0.738:0.738:64.594	45	90	80

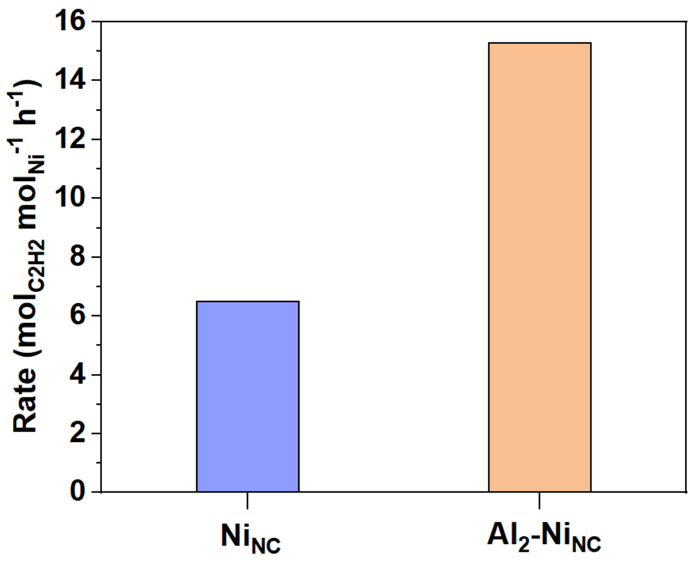

Supplementary Fig. 20 Catalytic performance. Reaction rate of Al_2-Ni_{NC}/NCNT and Ni nanoclusters

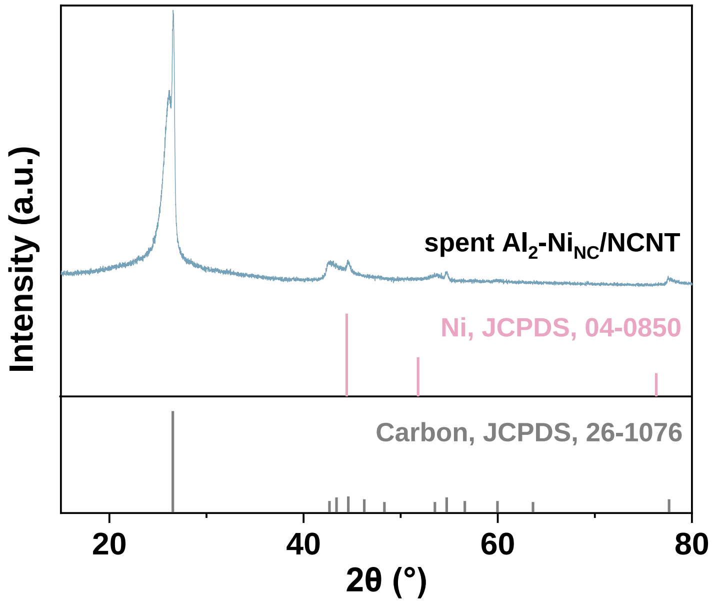

Supplementary Fig. 21 Analysis of crystal structure of spent Al_2-Ni_{NC}/NCNT derived from XRD pattern

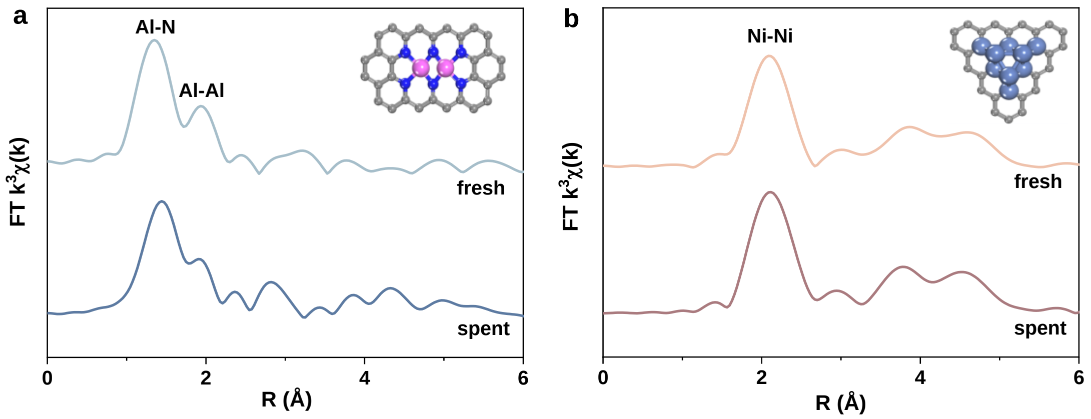

Supplementary Fig. 22 Analysis of coordination environment for Al and Ni species in spent catalysts. Fourier-transformed (a) Al and (b) Ni K-edge EXAFS spectra of spent Al_2-Ni_{NC}/NCNT compared with fresh ones (grey: C; blue: N; pink: Al; blue grey: Ni)

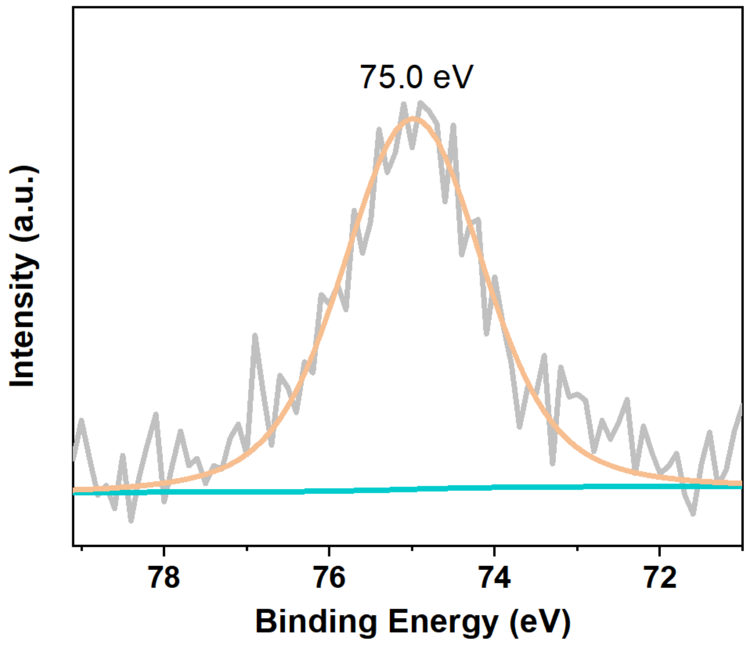

Supplementary Fig. 23 Analysis of electronic structure. Al 2*p* XPS spectra of spent Al_2-Ni_{NC}/NCNT

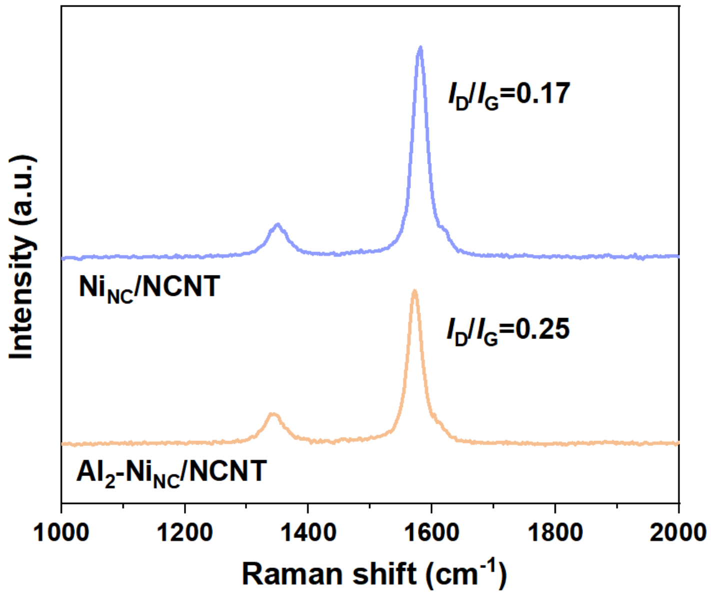

Supplementary Fig. 24 Raman spectra of spent Al_2-Ni_{NC}/NCNT and Ni_{NC}/NCNT to analyze the type of carbon species

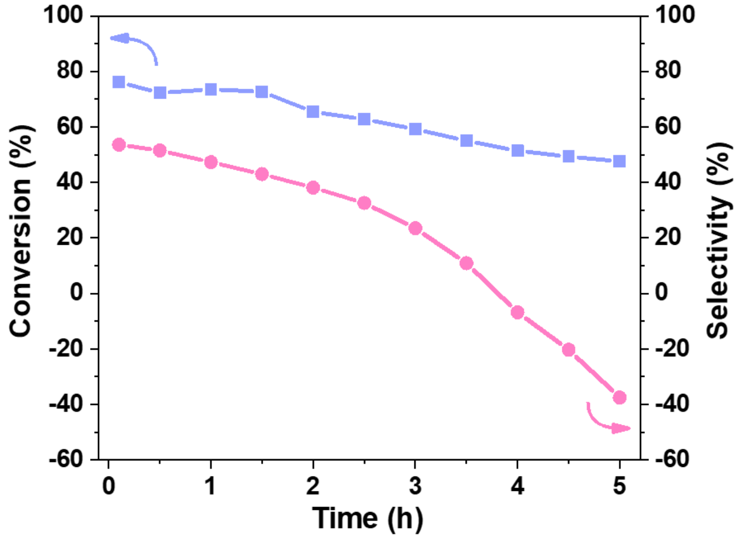

Supplementary Fig. 25 Durability test on Ni_{NC}/NCNT at 181 ℃ (Reaction condition: a hydrogen to acetylene ratio of 20:1; space velocity = 9000 mL g^{-1} h^{-1}; atmospheric pressure)

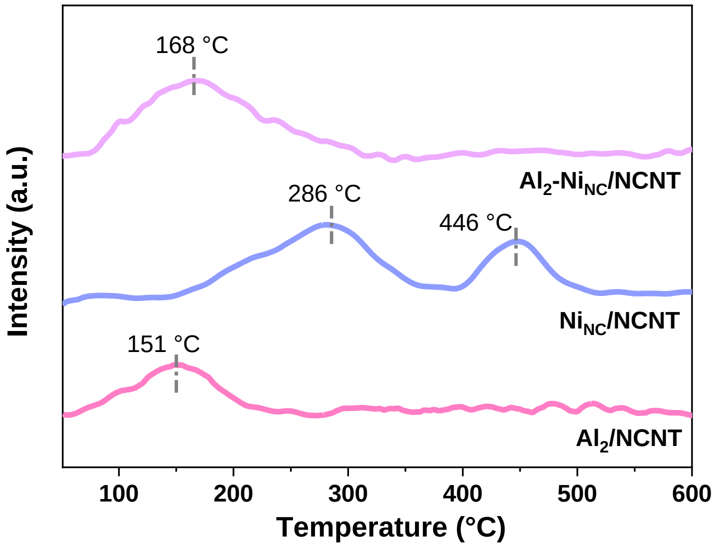

Supplementary Fig. 26 C_2H_4-TPD profiles on the different catalysts to obtain adsorption intensity and type of ethylene product

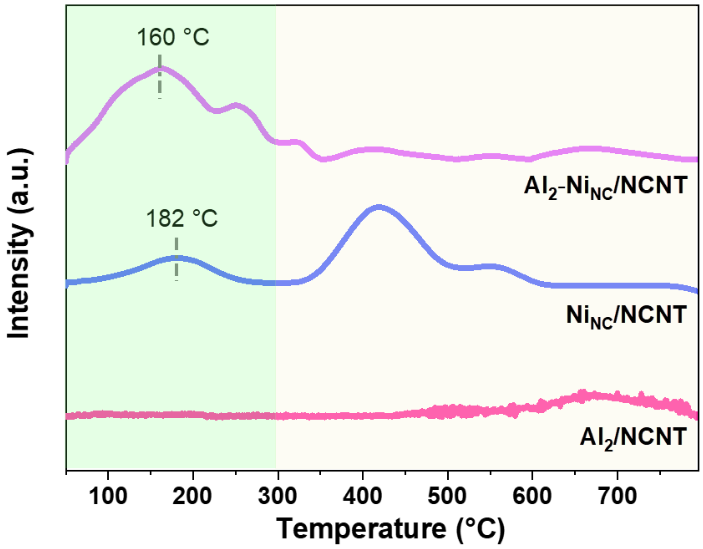

Supplementary Fig. 27 H_2-TPD profiles on the different catalysts to obtain adsorption intensity and type of reactant

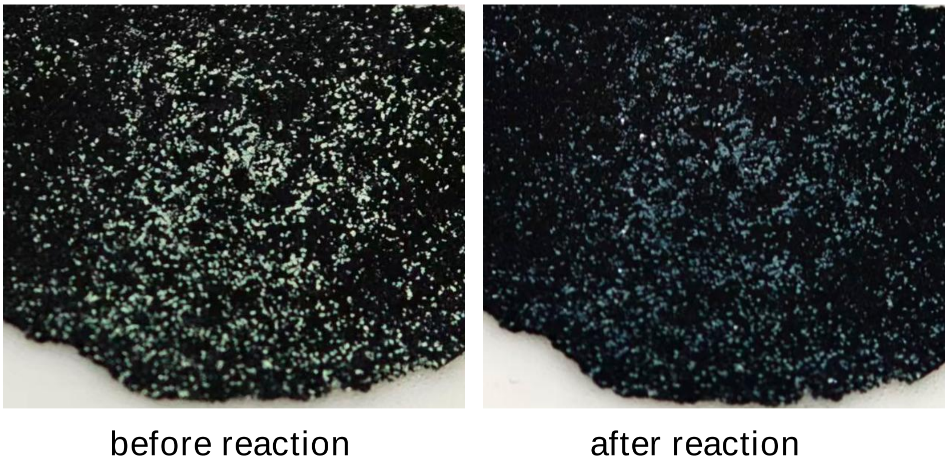

Supplementary Fig. 28 Verification of hydrogen spillover via color change of WO_3 and Al_2-Ni_{NC}/NCNT mixtures

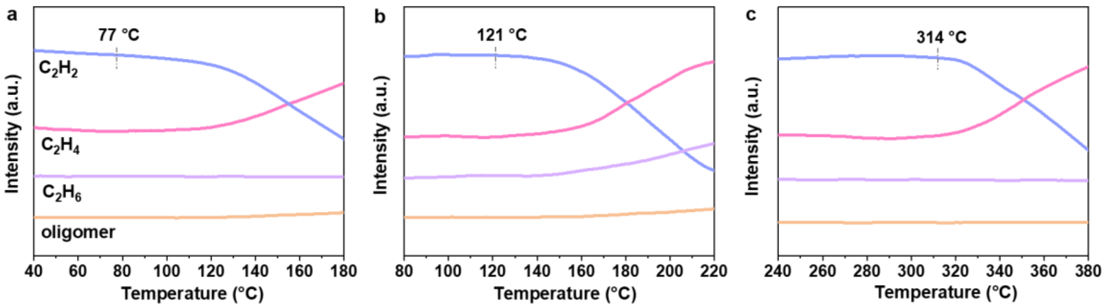

Supplementary Fig. 29 Surface reaction behavior. TPSR profiles of the acetylene reacting with hydrogen on the different catalysts. (a) Al_2-Ni_{NC}/NCNT, (b) Ni_{NC}/NCNT and (c) Al_2/NCNT

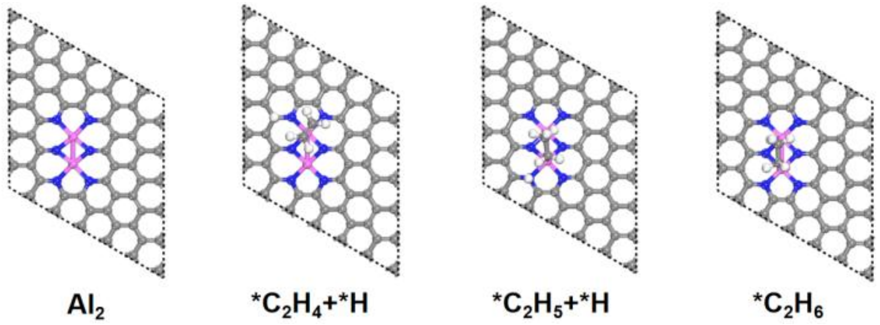

Supplementary Fig. 30 The different adsorption configurations on Al_2 sites

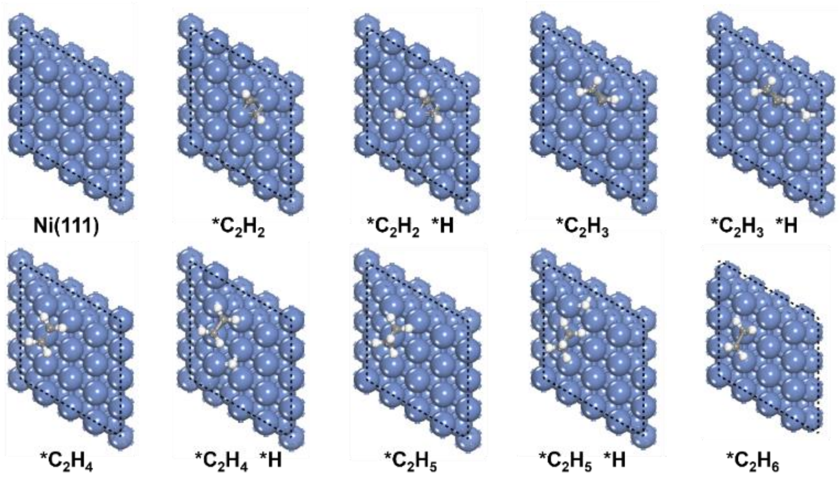

Supplementary Fig. 31 The different adsorption configurations of Ni (111)

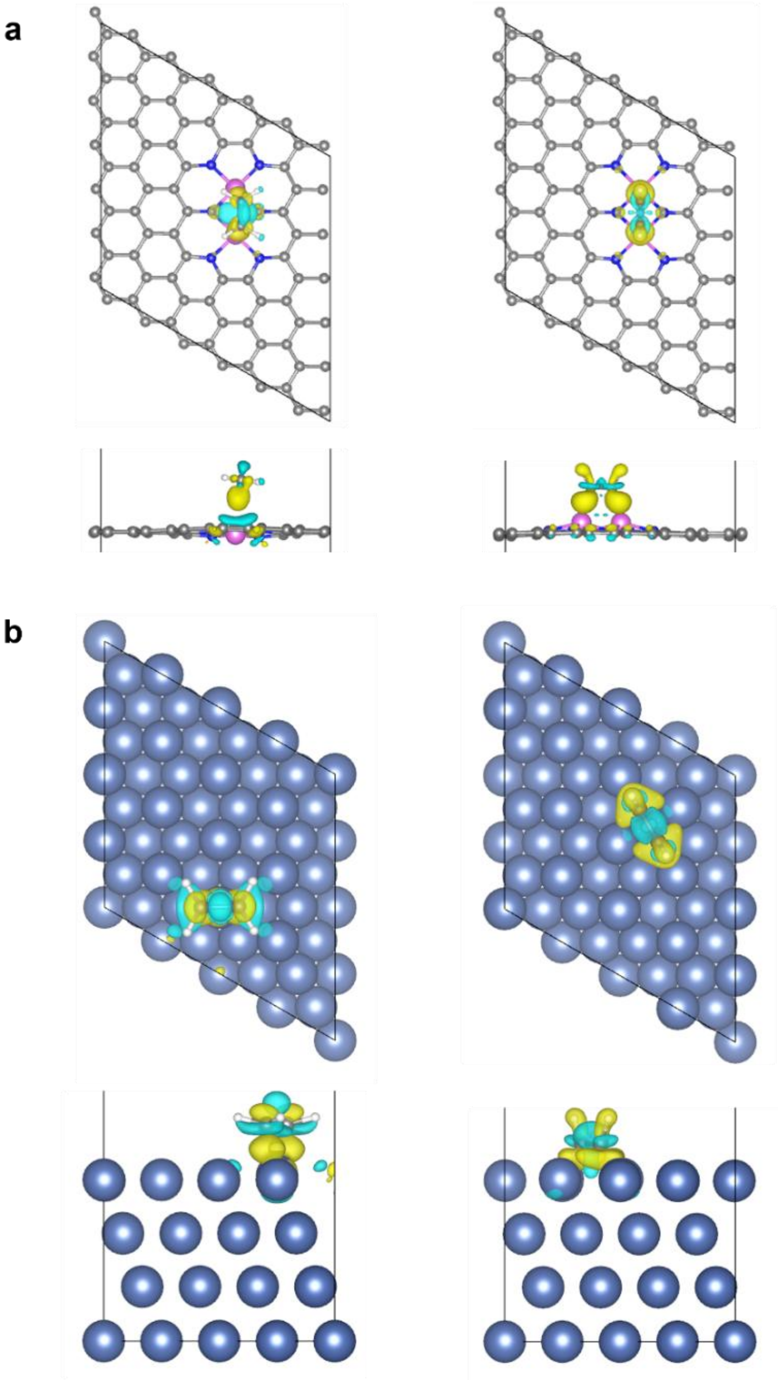

Supplementary Fig. 32 Differential charge density of C_2H_2 and C_2H_4 adsorbed on (a) Al_2 and (b) Ni (111). Yellow and cyan regions denote electron accumulation and depletion, respectively.

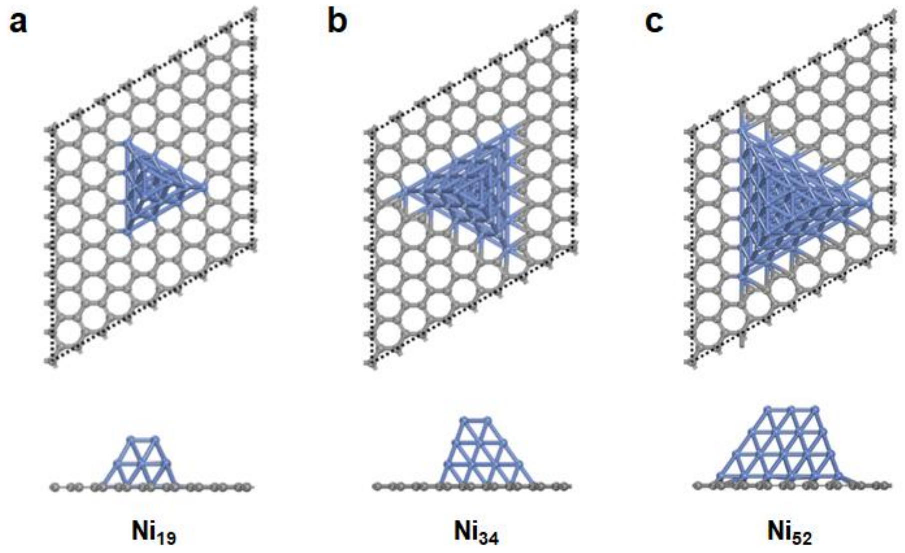

Supplementary Fig. 33 The structure models of (a) Ni_{19}, (b) Ni_{34}, and (c) Ni_{52} clusters from top and side views

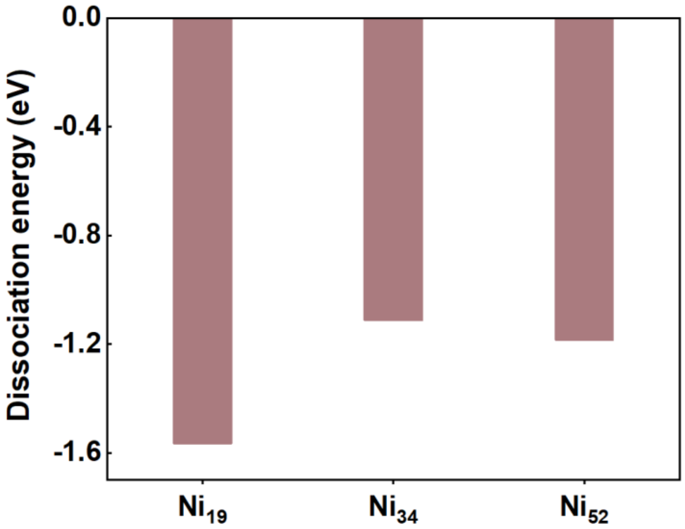

Supplementary Fig. 34 The dissociation energy of H_2 on Ni clusters

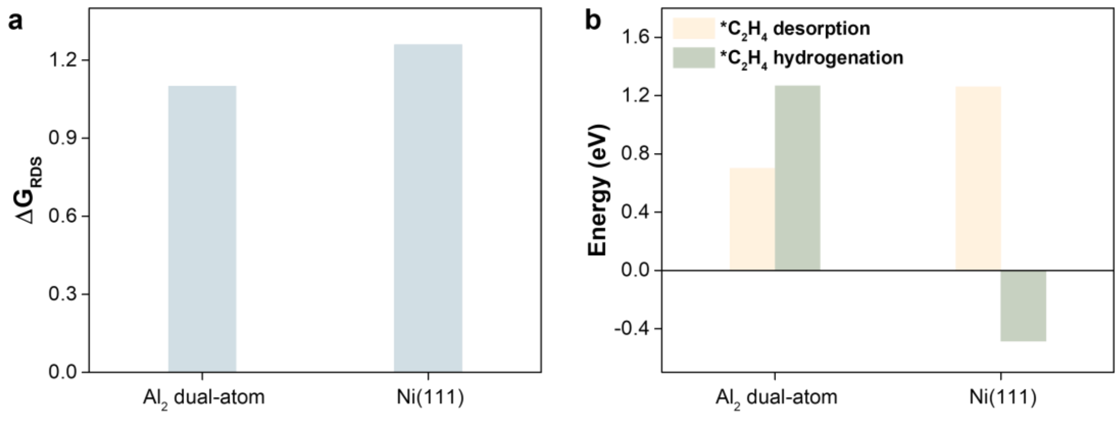

Supplementary Fig. 35 Reaction mechanism of acetylene hydrogenation. (a) The free energy of the rate-determining step (RDS) on the Al_2 dual-atom site and Ni (111) surface; (b) The desorption energy and hydrogenation energy of the C_2H_4 molecule on the Al_2 dual-atom site and Ni (111) surface

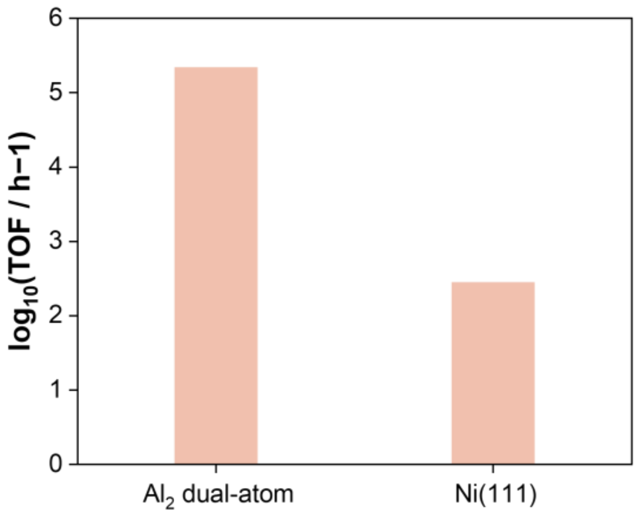

Supplementary Fig. 36 Reaction mechanism of acetylene hydrogenation. Logarithmic turnover frequencies (log (TOF)) of Ni (111) and Al_2 dual-atom sites at the reaction temperature of 430 K

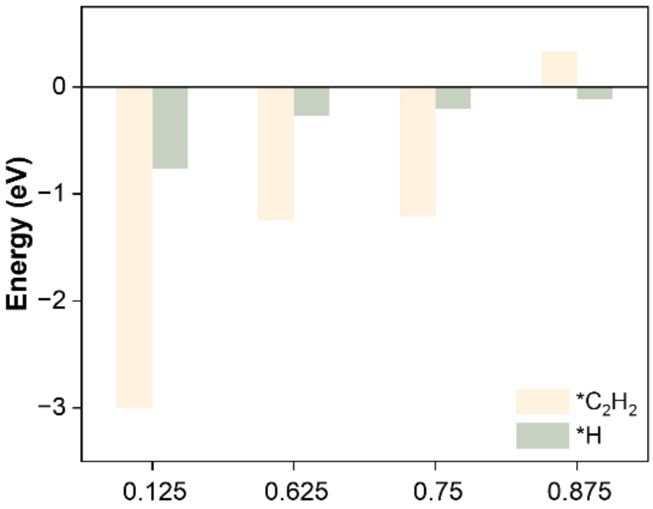

Supplementary Fig. 37 Reaction mechanism of acetylene hydrogenation. Adsorption energies of $*C_2H_2$ and *H on the Ni (111) surface under different C_2H_2 coverages

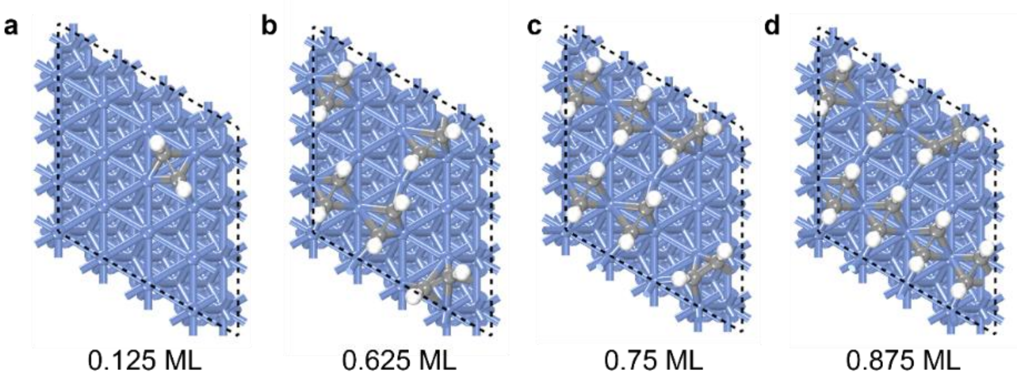

Supplementary Fig. 38 Atomic visualization of $*C_2H_2$ on the Ni (111) surface at different $*C_2H_2$ coverages

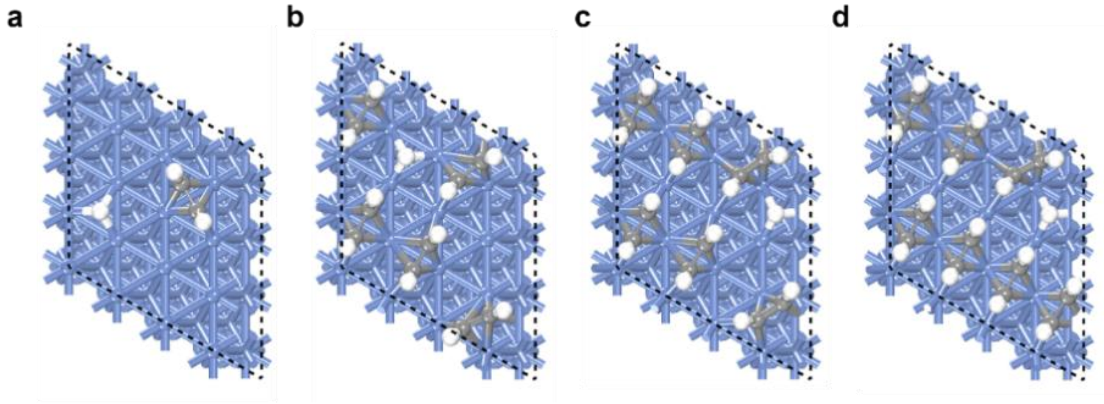

Supplementary Fig. 39 Atomic visualization of *H on the Ni (111) surface at different *C_2H_2 coverages

Supplementary References

1. Gu, J. et al. Synergizing metal-support interactions and spatial confinement boosts dynamics of atomic nickel for hydrogenations. *Nat. Nanotechnol.* **16**, 1141-1149 (2021).

2. Cao, Y. et al. Adsorption site regulation to guide atomic design of Ni-Ga catalysts for acetylene semi-hydrogenation. *Angew. Chem. Int. Ed*. **59**, 11647-11652 (2020).

3. Liu, Y. et al. Layered double hydroxide-derived Ni-Cu nanoalloy catalysts for semi-hydrogenation of alkynes: improvement of selectivity and anti-coking ability via alloying of Ni and Cu. *J. Catal*. **359**, 251-260 (2018).

4. Song, Y. et al. Understanding the Role of Coordinatively Unsaturated Al^{3+} Sites on Nanoshaped Al_2O_3 for Creating Uniform Ni-Cu Alloys for Selective Hydrogenation of Acetylene. *ACS Catal.* **13**, 1952-1963 (2023).

5. Riley, C. et al. Design of effective catalysts for selective alkyne hydrogenation by doping of ceria with a single-atom promotor. *J. Am. Chem. Soc*. **140**, 12964-12973 (2018).

6. Niu, Y. et al. Manipulating interstitial carbon atoms in the nickel octahedral site for highly efficient hydrogenation of alkyne. *Nat. Commun*. **11**, 1-9 (2020).

7. Liu, H. et al. Effect of IB-metal on Ni/SiO_2 catalyst for selective hydrogenation of acetylene. *Chin. J. Catal*. **41**, 1099-1108 (2020).

8. Zhou, H. et al. Sulfur-doped g-C_3N_4-supported Ni species with a wide temperature window for acetylene semi-hydrogenation. *ACS Sustain. Chem. Eng*. **10**, 4849-4861 (2022).

9. Li, Z. et al. Unveiling the origin of enhanced catalytic performance of NiCu alloy for semi-hydrogenation of acetylene. *Chem. Eng. J*. **450**, 138244 (2022).

10. Ma, J. et al. Nickel-based high-entropy intermetallic as a highly active and selective catalyst for acetylene semihydrogenation. *Angew. Chem. Int. Ed.* **134**, e202200889 (2022).

11. Sui, C. et al. Fully Exposed Nickel Clusters for Semihydrogenation of Acetylene. *ACS Catal.* **14**, 14689-14695 (2024).

12. Chai, Y. et al. Acetylene-selective hydrogenation catalyzed by cationic nickel confined in zeolite. *J. Am. Chem. Soc*. **141**, 9920-9927 (2019).

13. Fu, B. et al. Highly selective and stable isolated non-noble metal atom catalysts for selective hydrogenation of acetylene. *ACS Catal*. **12**, 607-615 (2021).

14. Ge, X. et al. Mechanism driven design of trimer Ni_1Sb_2 site delivering superior hydrogenation

selectivity to ethylene. *Nat. Commun*. **13**, 5534 (2022).

15. Yue, Y. et al. Tailoring Cu-Zn dual-atom sites with reordering d-orbital splitting manner for highly efficient acetylene semihydrogenation. *ACS Catal*. **14**, 3900-3911 (2024).

16. Huang, F. et al. Insight into the activity of atomically dispersed Cu catalysts for semihydrogenation of acetylene: impact of coordination environments. *ACS Catal*. **12**, 48-57 (2021).

17. Shi, X. et al. Copper catalysts in semihydrogenation of acetylene: from single atoms to nanoparticles. *ACS Catal*. **10**, 3495-3504 (2020) .

18. Huang, F. et al. Anchoring Cu_1 species over nanodiamond-graphene for semi-hydrogenation of acetylene. *Nat. Commun*. **10**, 4431 (2019).

19. Fu, F. et al. Interfacial bifunctional effect promoted non-noble Cu/Fe_yMgO_x catalysts for selective hydrogenation of acetylene. *ACS Catal*. **11**, 11117-11128 (2021).

20. Pei, G. et al. Performance of Cu-alloyed Pd single-atom catalyst for semihydrogenation of acetylene under simulated front-end conditions. *ACS Catal.* **7**, 1491-1500 (2017).

21. Zhou, S. et al. Bi-modified Cu-based catalysts for acetylene hydrogenation: leveraging dispersion and hydrogen spillover. *Inorg. Chem*. **63**, 11802-11811 (2024).

22. Lu, C. et al. Copper-based catalysts for selective hydrogenation of acetylene derived from $Cu(OH)_2$. *ACS omega*. **6**, 3363-3371 (2021).

23. Liu, T. et al. Hydrophobic surface modification of Cu-based catalysts for enhanced semihydrogenation of acetylene in excess ethylene. *ACS Catal.* **14**, 5838-5846 (2024).

24. Li, Z. et al. Unveiling the origin of enhanced catalytic performance of NiCu alloy for semi-hydrogenation of acetylene. *Chem. Eng. J*. **450**, 138244 (2022).

25. Tejeda-Serrano, M. et al. Isolated Fe (III)-O sites catalyze the hydrogenation of acetylene in ethylene flows under front-end industrial conditions. *J. Am. Chem. Soc*. **140**, 8827-8832 (2018).

26. Xue, F. et al. Atomic three-dimensional investigations of Pd nanocatalysts for acetylene semi-hydrogenation. *J. Am. Chem. Soc*. **145**, 26728-26735 (2023).

27. Huang, F. et al. Low-temperature acetylene semi-hydrogenation over the Pd_1-Cu_1 dual-atom catalyst. *J. Am. Chem. Soc.* **144**, 18485-18493 (2022).

28. Guo, Y. et al. Photo-thermo semi-hydrogenation of acetylene on Pd_1/TiO_2 single-atom catalyst. *Nat. Commun*. **13**, 2648 (2022).

29. Ballesteros-Soberanas, J. et al. A MOF-supported Pd_1-Au_1 dimer catalyses the

semihydrogenation reaction of acetylene in ethylene with a nearly barrierless activation energy. *Nat Catal*. **7**, 452-463 (2024).

30. Liu, Y. et al. Polyoxometalate-based metal-organic framework as molecular sieve for highly selective semi-hydrogenation of acetylene on isolated single Pd atom sites. *Angew. Chem. Int. Ed.* **60**, 22522-22528 (2021).

31. Dasgupta, A. et al. Atomic control of active-site ensembles in ordered alloys to enhance hydrogenation selectivity. *Nat. Chem*. **14**, 523-529 (2022).

32. Zou, S. et al. Grafting nanometer metal/oxide interface towards enhanced low-temperature acetylene semi-hydrogenation. *Nat. Commun*. **12**, 5770 (2021) .

33. Zhou, S.et al. Pd single-atom catalysts on nitrogen-doped graphene for the highly selective photothermal hydrogenation of acetylene to ethylene. *Adv. Mater.* **31**, 1900509 (2019).

34. Gao, Q. et al. Atomic layers of B_2 CuPd on Cu nanocubes as catalysts for selective hydrogenation. *J. Am. Chem. Soc*. **145**, 19961-19968 (2023).

35. Huang, F. et al. Atomically dispersed Pd on nanodiamond/graphene hybrid for selective hydrogenation of acetylene. *J. Am. Chem. Soc*. **140**, 13142-13146 (2018).

36. Li, J. et al. Stable and size-controllable ultrafine Pt nanoparticles derived from a MOF-based single metal ion trap for efficient electrocatalytic hydrogen evolution. *J. Mater. Chem. A*. **7**, 20239-20246 (2019).

37. Feng, Q. et al. Isolated single-atom Pd sites in intermetallic nanostructures: high catalytic selectivity for semihydrogenation of alkynes. *J. Am. Chem. Soc*. **139,** 7294-7301 (2017).

38. Wang, S. et al. Activation and spillover of hydrogen on sub-1 nm palladium nanoclusters confined within sodalite zeolite for the semi-hydrogenation of alkynes. *Angew. Chem. Int. Ed*. **58**, 7668-7672 (2019).

39. Zhang, L. et al. Pd@C core-shell nanoparticles on carbon nanotubes as highly stable and selective catalysts for hydrogenation of acetylene to ethylene. *Nanoscale*. 9, 14317-14321 (2017).

40. Li, R. et al. Selective hydrogenation of acetylene over Pd-Sn catalyst: Identification of Pd_2Sn intermetallic alloy and crystal plane-dependent performance. *Appl. Catal. B Environ*. **279**, 119348 (2020).

41. McCue, A. J. et al. Palladium sulphide-A highly selective catalyst for the gas phase hydrogenation of alkynes to alkenes. *J. Catal*. **340**, 10-16 (2016).

42. Riyapan, S. et al. Preparation of improved Ag-Pd/TiO_2 catalysts using the combined strong electrostatic adsorption and electroless deposition methods for the selective hydrogenation of acetylene. *Catal. Sci. Technol*. **6**, 5608-5617 (2016).

43. Wu, P. et al. Harnessing strong metal-support interactions via a reverse route. *Nat. Commun*. **11**, 3042 (2020).

44. Feng, Q. et al. Mesoporous nitrogen-doped carbon-nanosphere-supported isolated single-atom Pd catalyst for highly efficient semihydrogenation of acetylene. *Adv. Mater*. **31**, 1901024 (2019).

45. Wei, S. et al. Direct observation of noble metal nanoparticles transforming to thermally stable single atoms. *Nat. Nanotechnol*. **13**, 856-861 (2018).

46. Huang, X. et al. Enhancing both selectivity and coking-resistance of a single-atom Pd_1/C_3N_4 catalyst for acetylene hydrogenation. *Nano Res*. **10**, 1302-1312 (2017).

47. Hu, M. et al. 50 ppm of Pd dispersed on $Ni(OH)_2$ nanosheets catalyzing semi-hydrogenation of acetylene with high activity and selectivity. *Nano Res*. **11**, 905-912 (2018).

48. Tao, X. et al. Highly active isolated single-atom Pd catalyst supported on layered MgO for semihydrogenation of acetylene. *ACS Appl. Energy Mater.* **5**, 10385-10390 (2022).

49. Guo, Y. et al. High Performance of Single-atom Catalyst Pd_1/MgO for Semi-hydrogenation of Acetylene to Ethylene in Excess Ethylen. *ChemNanoMat*. **7**, 526-529 (2021).

50. Zhou, H. et al. Pd/ZnO catalysts with different origins for high chemoselectivity in acetylene semi-hydrogenation. *Chin. J. Catal*. **37**, 692-699 (2016).

51. Guo, Y. et al. Pd single-atom catalysts derived from strong metal-support interaction for selective hydrogenation of acetylene. *Nano Res*. **15**, 10037-10043 (2022).

52. Li, Z. et al. Regulating metal-support interactions of Pd/$MgAl_2O_4$ for efficient selective hydrogenation of acetylene. *Catal. Today*. **423**, 114253 (2023).

53. Hu, M. et al. N_8 stabilized single-atom Pd for highly selective hydrogenation of acetylene. *J. Catal*. **395**, 46-53 (2021).

54. Li, R. et al. Graphdiyne anchoring to construct highly dense palladium trimer active sites for the selective hydrogenation of acetylene. *Nano Res*. **16**, 6167-6177 (2023).

55. Zhou, H. et al. PdZn intermetallic nanostructure with Pd-Zn-Pd ensembles for highly active and chemoselective semi-hydrogenation of acetylene. *ACS Catal.* **6**, 1054-1061 (2016).

56. Dehghani, O., Rahimpour, M. & and Shariati, A. An experimental approach on industrial Pd-

Ag supported α-Al_2O_3 catalyst used in acetylene hydrogenation process: mechanism, kinetic and catalyst decay. *Processes*. **7**, 136 (2019).
